# Supplementary material for: Influenza A Virus Infection Reactivates Human Endogenous Retroviruses Associated with Modulation of Antiviral Immunity
Source: Viruses. 2022 Jul 21;14(7):1591. doi: 10.3390/v14071591 (PMC9320126; doi:10.3390/v14071591)
Supplement: Supplementary file 1 [file viruses-14-01591-s001.zip › MotifAnalysis.zip!/MotifOutput_SC35M/homerResults/motif15.info.html]

Motif 15

## Information for 14-GRARKRCCTGRY (Motif 15)

A
C
T
G
C
T
G
A
C
G
T
A
C
T
G
A
A
C
G
T
C
T
G
A
A
G
T
C
A
G
T
C
A
C
G
T
A
C
T
G
C
T
A
G
A
G
C
T
  
Reverse Opposite:
